# Identification and validation of key biomarkers for the early diagnosis of diabetic kidney disease

**DOI:** 10.3389/fphar.2022.931282

**Published:** 2022-08-22

**Authors:** Wei Yu, Ting Wang, Feng Wu, Yiding Zhang, Jin Shang, Zhanzheng Zhao

**Affiliations:** ^1^ Department of Nephrology, The First Affiliated Hospital of Zhengzhou University, Zhengzhou, China; ^2^ Institute of Nephrology, Zhengzhou University, Zhengzhou, China; ^3^ Laboratory Animal Platform of Academy of Medical Sciences, Zhengzhou University, Zhengzhou, China; ^4^ Laboratory of Nephrology, The First Affiliated Hospital of Zhengzhou University, Zhengzhou, China

**Keywords:** key biomarkers, diabetic kidney disease, diagnostic model, differential genes, immune infiltration

## Abstract

**Background:** Diabetic kidney disease (DKD) is the leading cause of end-stage renal disease. This study explored the core genes and pathways associated with DKD to identify potential diagnostic and therapeutic targets.

**Methods:** We downloaded microarray datasets GSE96804 and GSE104948 from the Gene Expression Omnibus (GEO) database. The dataset includes a total of 53 DKD samples and 41 normal samples. Differentially expressed genes (DEGs) were identified using the R package “limma”. The Metascape database was subjected to Gene Ontology (GO) function and Kyoto Encyclopedia of Genes and Genomes (KEGG) pathway enrichment analyses to identify the pathway and functional annotations of DEGs. A WGCAN network was constructed, the hub genes in the turquoise module were screened, and the core genes were selected using LASSO regression to construct a diagnostic model that was then validated in an independent dataset. The core genes were verified by *in vitro* and *in vivo* experiments.

**Results:** A total of 430 DEGs were identified in the GSE96804 dataset, including 285 upregulated and 145 downregulated DEGs. WGCNA screened out 128 modeled candidate gene sets. A total of eight genes characteristic of DKD were identified by LASSO regression to build a prediction model. The results showed accuracies of 99.15% in the training set (GSE96804) and 94.44% and 100%, respectively, in the test (GSE104948-GPL22945 and GSE104948-GPL24120). Three core genes (*OAS1, SECTM1,* and *SNW1*) with high connectivity were selected among the modeled genes. *In vitro* and *in vivo* experiments confirmed the upregulation of these genes.

**Conclusion:** Bioinformatics analysis combined with experimental validation identified three novel DKD-specific genes. These findings may advance our understanding of the molecular basis of DKD and provide potential therapeutic targets for its clinical management.

## 1 Introduction

Diabetic kidney disease (DKD) is one of the most common microvascular complications of diabetes and the leading cause of end-stage renal disease (ESRD) worldwide (Alicic et al., 2017). The main lesion of DKD is located in the glomeruli and is characterized by extracellular matrix deposition and thickening of the basement membrane (Wada and Makino, 2013). The etiology and pathogenesis of DKD are not yet fully understood. The early diagnosis of DKD has traditionally been made based on the presence of microalbuminuria (MA) and the course of diabetes mellitus (DM) (Papadopoulou-Marketou et al., 2017). However, this method is not accurate since only 30% of cases were confirmed by pathology, while others were primary glomerular diseases superimposed on DM (Oh et al., 2012). Therefore, the early diagnosis of DKD based on clinical indicators is insufficient and biomarkers are lacking.

In recent decades, an increasing number of biomarkers for DKD have been reported (Levey et al., 2011; Van et al., 2017). Bioinformatics technology has also been used to identify biomarkers that are closely related to disease progression and to find new targets for the early diagnosis and treatment of diseases (Udhaya Kumar et al., 2020).

The present study analyzed the GEO database (GSE96804, GSE104948 datasets) to find potential biomarkers for DKD. We also constructed diabetic nephropathy models in mice and mesangial cells. We further performed *in vivo* and *in vitro* experiments to verify the reliability of the core genes as DKD biomarkers. The results of this study may provide new clues for the diagnosis and potential therapeutic targets of DKD.

## 2 Materials and methods

### 2.1 Data download

We downloaded the Series Matrix Files of GSE96804 and GSE104948 from the GEO database (http://www.ncbi.nlm.nih.gov/geo). GSE96804 included 41 DKD kidney tissue samples and 20 control samples based on the GPL1261 annotation platform. GSE104948 contains two annotation platform files. GSE104948-GPL22945 included seven DKD kidney tissue samples and 18 control samples. GSE104948-GPL24120 included five DKD kidney tissue samples and three control samples. Based on the above datasets, this study included a total of 53 DKD and 41 healthy control glomerular samples. Differential analysis was performed using the R package “limma” to explore the differences in the molecular mechanisms of DKD.

### 2.2 Functional annotation using Gene ontology and Kyoto Encyclopedia of Genomes analyses

Differential genes were functionally annotated using the Metascape database (http://metascape.org) to comprehensively explore their functional relevance. Gene ontology (GO) and Kyoto Encyclopedia of Genomes (KEGG) pathway analyses were performed on specific genes. A minimum overlap ≥3 and *p* ≤ 0.01 were considered statistically significant.

### 2.3 Model building

After identifying differential genes, LASSO regression was used to further construct a prediction model. After incorporating the expression values for each gene, a scoring formula for each patient was constructed and weighted by its estimated regression coefficients in a LASSO regression analysis. According to the scoring formula, the median risk score value was used as the cut-off point. The patients were divided into high-scoring and low-scoring groups. ROC curves were used to assess the accuracy of the model prediction.

### 2.4 Construction of WGCNA co-expression network

We constructed a weighted gene co-expression network to search for co-expressed gene modules. We further explored the relationships between gene networks, phenotypes, and the core genes. Co-expression networks of differential genes in the GSE96804 dataset were constructed using the WGCNA-R package, with the soft threshold set to eight (Chen et al., 2019). The weighted adjacency matrix was transformed into a topological overlap matrix (TOM) to estimate the degree of network connectivity. The hierarchical clustering method was used to construct the clustering tree structure of the TOM matrix. Different branches and colors of the clustering tree represented different gene modules. Genes were classified according to their expression patterns based on their weighted correlation coefficients. Genes with similar patterns were grouped into a module such that all genes were divided into multiple modules based on the gene expression patterns.

### 2.5 Gene ontology semantic similarity

Based on the similarity in GO semantics for gene annotation, we ranked proteins according to the functional similarities between them and their interacting partners. GO semantic similarity has been validated by correlation with gene expression profiles, providing a basis for the functional comparison of gene products. Thus, it has been widely used in bioinformatics, such as in protein-protein interaction analysis, pathway analysis, and gene function prediction. In the present study, we measured the functional similarity between proteins. Functional similarity was defined as the geometric mean of the semantic similarity of GO in terms of molecular function (MF) and biological pathway (BP). The aim was to measure the strength of the relationship between each protein and its interacting proteins by considering the function and pathway. Semantic similarity between interacting histones in MF and BP was assessed by using the GOSe disease im package, which was performed more accurately by considering the GO topology (Wang et al., 2007). Functional similarity was further estimated based on the geometric mean of semantic similarity in MF and BP.

### 2.6 Analysis of immune cell infiltration

CIBERSORT is a widely used evaluation method for immune cell types in the micro-environment. This method is based on the principle of support vector regression to perform a deconvolution analysis of the expression matrix of immune cell subtypes. CIBERSORT contains 547 biomarkers that distinguish 22 human immune cell phenotypes, including T, B, plasma, and myeloid subsets. In this study, we applied the CIBERSORT algorithm to analyze patient data to infer the relative proportions of the 22 types of immune infiltrating cells and to perform Spearman correlation analyses of gene expression and immune cell content.

### 2.7 GSEA analysis

GSEA analysis uses a predefined set of genes. It ranks genes according to their degree of differential expression in two types of samples and then tests whether the predefined gene set is enriched at the top or bottom of the ranking list. In this study, GSEA was used to compare the differences in the KEGG signaling pathway between the high expression group and the low expression group, and to explore the molecular mechanism of the core genes in the two groups of patients. The number of substitutions was set to 1,000, and the substitution type was set to phenotype.

### 2.8 Competing endogenous RNA network

Competing endogenous RNA (ceRNA) has attracted research attention in recent years. They represent a completely new mode of gene expression regulation. Compared to the miRNA regulatory network, the ceRNA regulatory network is more elaborate and complex. It involves many more RNA molecules, including mRNA, pseudogenes encoding genes, miRNAs, lncRNAs, etc. The NPInter database is commonly used to query the relationship between lncRNAs and miRNAs. We used the NPInter database to predict lncRNA-miRNA interaction pairs. In addition, we also used the combined miRcode database to back-predict mRNA-miRNA interactions. A lncRNA-miRNA-mRNA network was then established by combining lncRNA-miRNA and mRNA-miRNA interactions and visualized using Cytoscape.

### 2.9 Experimental animals and procedures

Male C57BL/6 mice (6–8 weeks) were used to construct the diabetes model. Each group included five mice. The grouping was randomized.

The mouse model of diabetes was induced by intraperitoneal injection of STZ (Sigma-Aldrich) at 50 mg/kg body weight in 100 mmol/L sodium citrate (pH 4.5) for five consecutive days (*n* = 5). All mice were euthanized at the 20th week, the fasting blood glucose levels of all groups were measured, and 24 h urine and kidney tissue were collected. All animal experimental protocols were approved by the Animal Care and Use Committee of Zhengzhou University (2019-KY-43).

### 2.10 Cell culture

Human mesangial cells (HMC) were purchased from the American Type Culture Collection. Cells were cultured in DMEM medium (Gibco) containing 10% fetal bovine serum (FBS, Gibco) at 37°C in a 5% CO_2_ atmosphere. HMCs were cultured in normal glucose (5.5 mmol/L) or high D-glucose (40 mmol/L) medium for 24 h.

### 2.11 Immunohistochemical staining

Immunohistochemical staining was performed using standard protocols. Sections were prepared for immunohistochemistry with primary antibodies for rabbit polyclonal anti-SNW1 (ab167165, Abcam), anti-OAS1 (14955-1-AP, Proteintech), anti-SECTM1 (bs-10153R, Bioss), anti-CD4 (ab183685, Abcam), and secondary antibodies for Goat anti-Rabbit IgG.

### 2.12 RNA extraction and quantitative RT-PCR

RNA was extracted using Takara RR420 and RR036A isolation kits according to the manufacturer’s instructions. First-strand cDNA synthesis was performed using PrimeScript reverse transcriptase (Takara RR420). qPCR was performed using the Takara RR036A kit. β-Actin was used as an internal reference. The relative expression levels of the target genes were calculated using the 2^−ΔΔCt^ method. A *p*-value < 0.05 was considered significant. The primer sequences are summarized in Supplementary Table S1. All PCR reactions were conducted in triplicate.

### 2.13 Western blot analysis

Total proteins were extracted from cells and tissues using RIPA buffer. Proteins of different molecular weights were separated by SDS-PAGE. The proteins were then electrotransferred onto PVDF membranes, which were placed in 5% nonfat milk and blocked for 1 h at room temperature before incubation with specific primary antibodies, including anti-SNW1 (ab167165, Abcam), anti-OAS1 (14955-1-AP, Proteintech), anti-SECTM1 (bs-10153R, Bioss) overnight at 4°C. The membranes were then incubated with secondary antibody (ab205718, Abcam) for 1.5 h at room temperature. The protein bands were visualized and analyzed using enhanced chemiluminescence reagents (UElandy) and ImageJ (National Institutes of Health, V1.8.0).

### 2.14 Statistical analysis

All statistical analyses were performed in R (version 3.6). All statistical tests were two-sided and *p* < 0.05 was considered statistically significant.

## 3 Results

### 3.1 Identification of differentially expressed genes and functional enrichment in diabetic kidney disease

**FIGURE 1:**
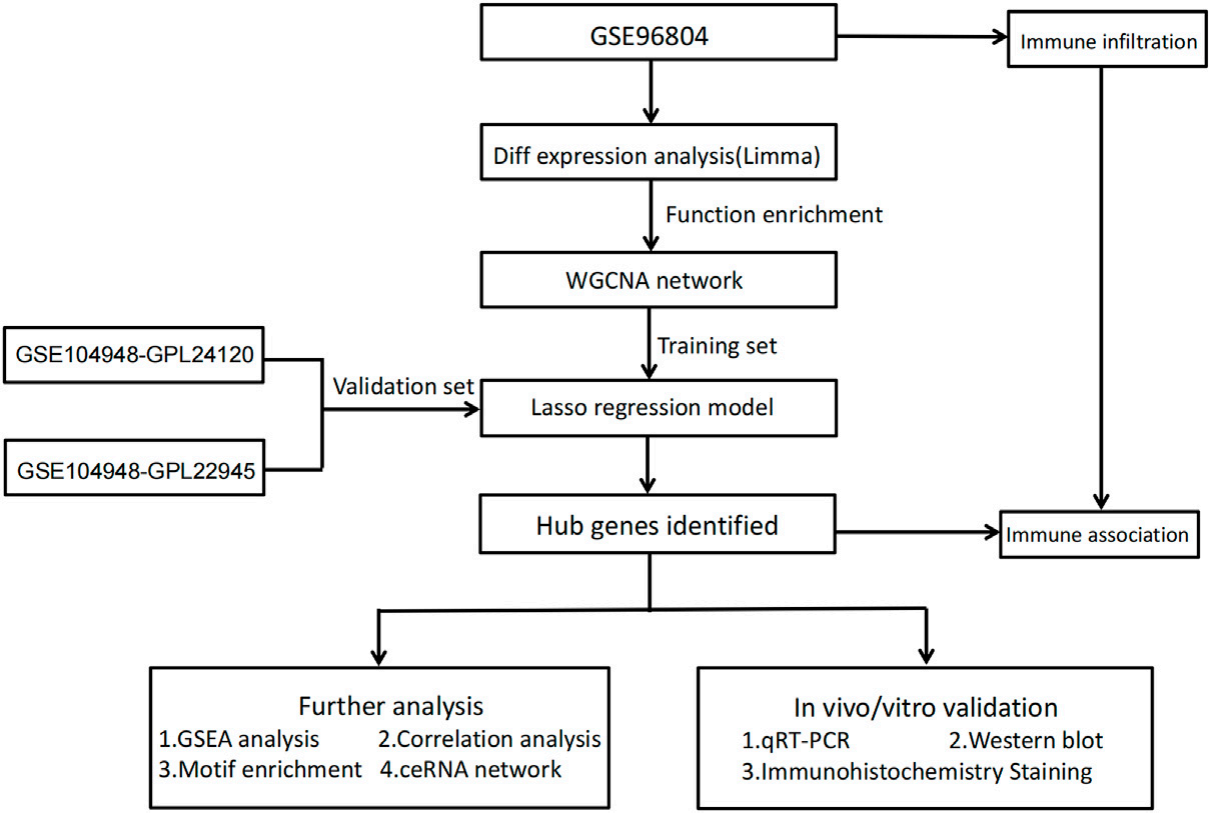
Study workflow.

**FIGURE 2:**
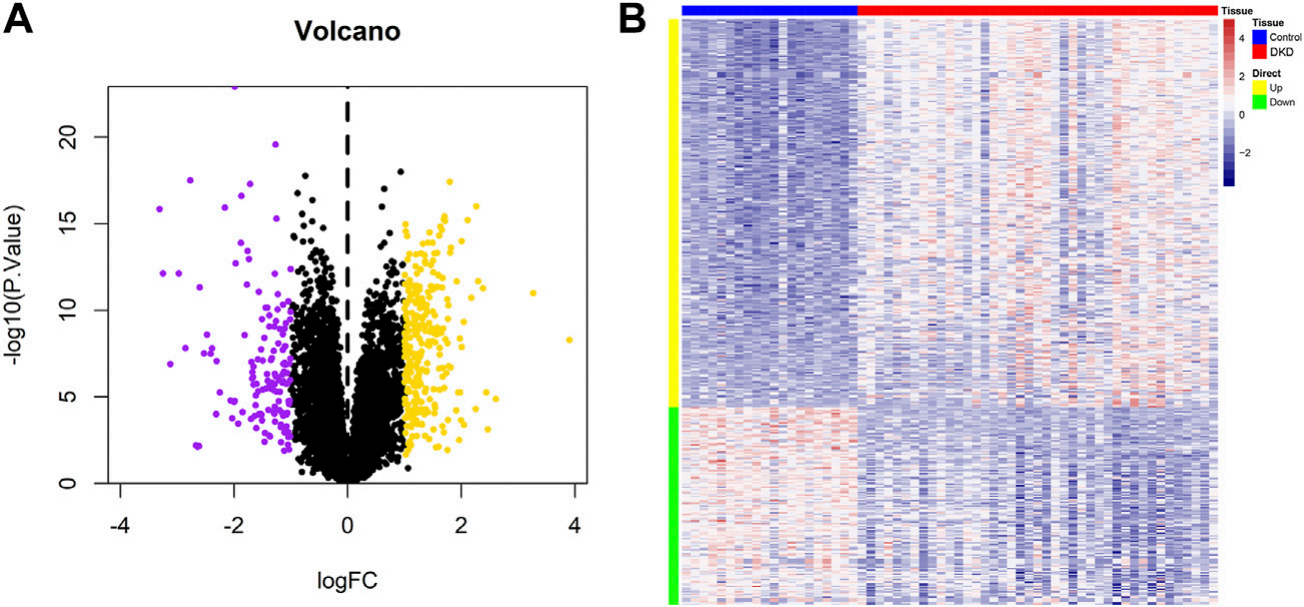
Identification of differential genes in DKD. **(A)** Volcano plot of the differential expression of GSE96804. Purple and yellow indicate the down-regulation and upregulation of differential expression, respectively (screening conditions: *p* < 0.05 and |Log2FC|>0.585). **(B)** Heatmap of differential gene expression.

**FIGURE 3:**
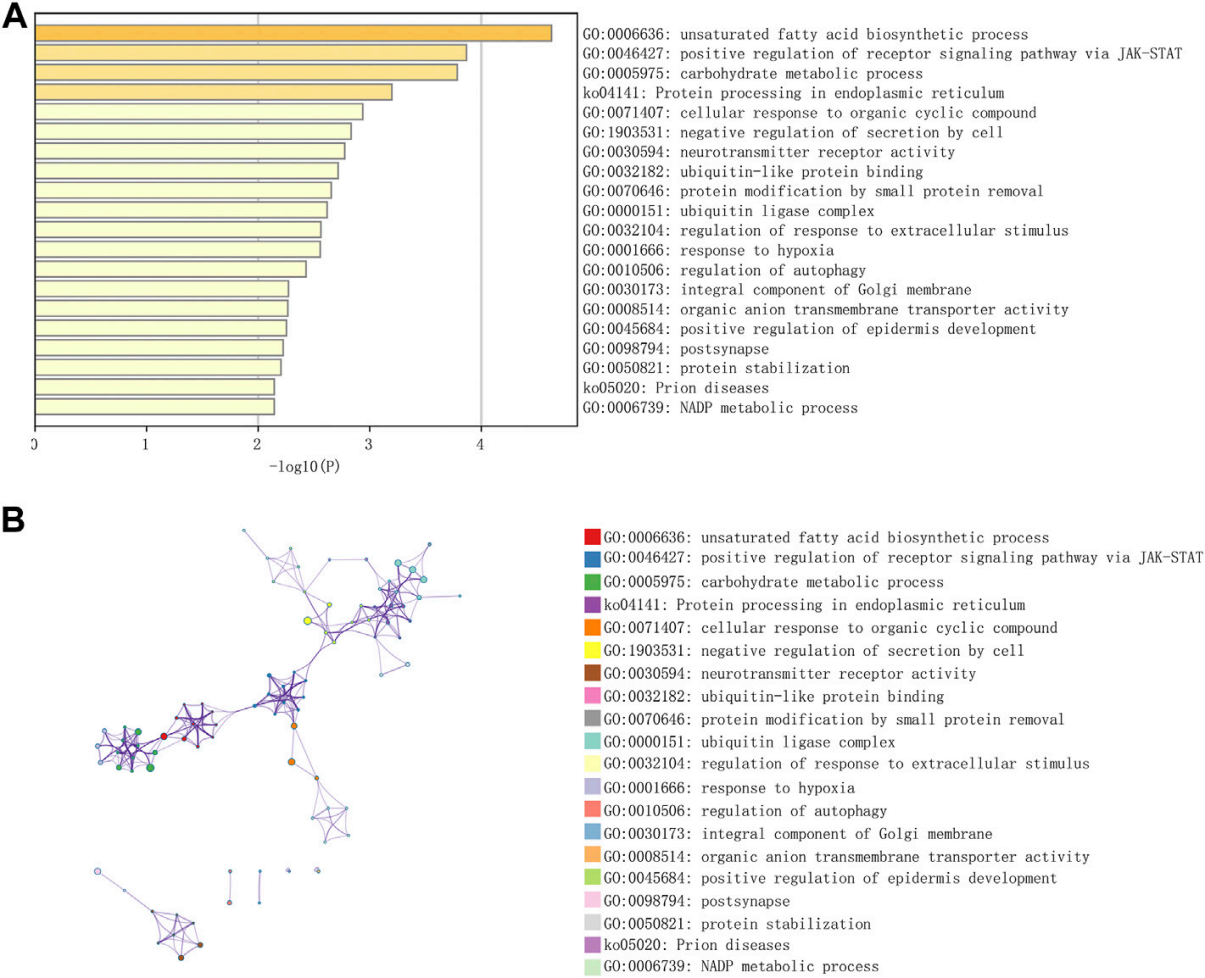
Functional enrichment of differential genes in DKD. **(A)** GO-KEGG enrichment analysis of differential genes from the Metascape database. **(B)** A cluster network of enriched pathways, in which nodes that share the same cluster are often located close to each other.

Figure 1 shows the workflow of the present study. We downloaded the GSE96804 dataset from the NCBI GEO public database. The 61 groups included 20 in the normal group and 41 in the DKD group. We used the limma package to calculate the differential genes between the two groups (*p* < 0.05 and |Log2FC|>1). A total of 430 differential genes were screened, including 285 upregulated genes and 145 downregulated genes, as shown by the Volcano map and differential gene heatmap (Figures 2A, B). Enrichment analysis of these 430 differential genes through the Metascape online database showed that these genes were mainly enriched in unsaturated fatty acid biosynthetic process, positive regulation of receptor signaling pathway via JAK-STAT, carbohydrate metabolic processing, protein processing in the endoplasmic reticulum, and prion diseases (Figures 3A, B).

### 3.2 WGCNA co-expression network analysis

**FIGURE 4:**
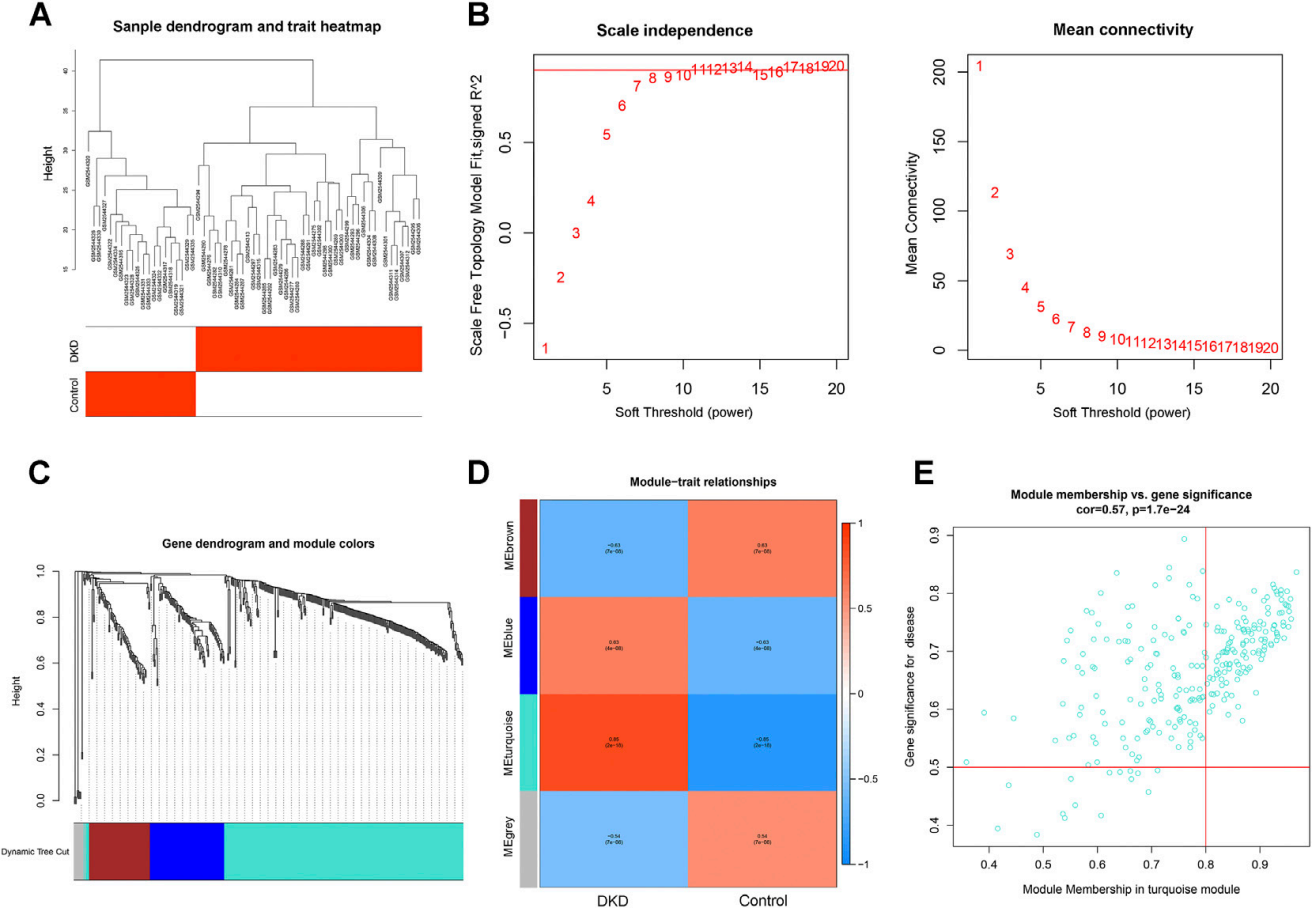
Construction of a WGNCA network. **(A)** Clustering heatmap of control and DKD samples. **(B)** Scale-free exponent and average connectivity for each soft threshold. **(C)** Dendrogram of gene clusters, with different colors representing different modules. **(D)** Heatmap of the correlations between module eigengenes and DKD. Blue and red indicate negative and positive correlations, respectively. The turquoise module with the highest correlation was selected for subsequent analysis. **(E)** Scatter plot of genes in the turquoise module. The hub genes in the turquoise module were screened according to the criteria of |MM|>0.8 and |GS|>0.5.

To identify the key genes in the differential gene set, we constructed a WGCNA network based on the differential genes in GSE96804. The soft threshold was set to 8, as determined by the “sft$powerEstimate” function. The gene modules were then detected based on the tom matrix. A total of four gene modules were detected: blue (82), brown (67), grey (12), and turquoise (268). The turquoise module showed the highest correlation [cor = 0.85, p = (2e−18)]. We screened the hub genes in the turquoise module based on the criteria of |MM|>0.8 and |GS|>0.5. A total of 128 genes were eligible and were modeled as a candidate gene set (Figures 4A–E).

### 3.3 LASSO model to identify potential predictive markers of diabetic kidney disease

**FIGURE 5:**
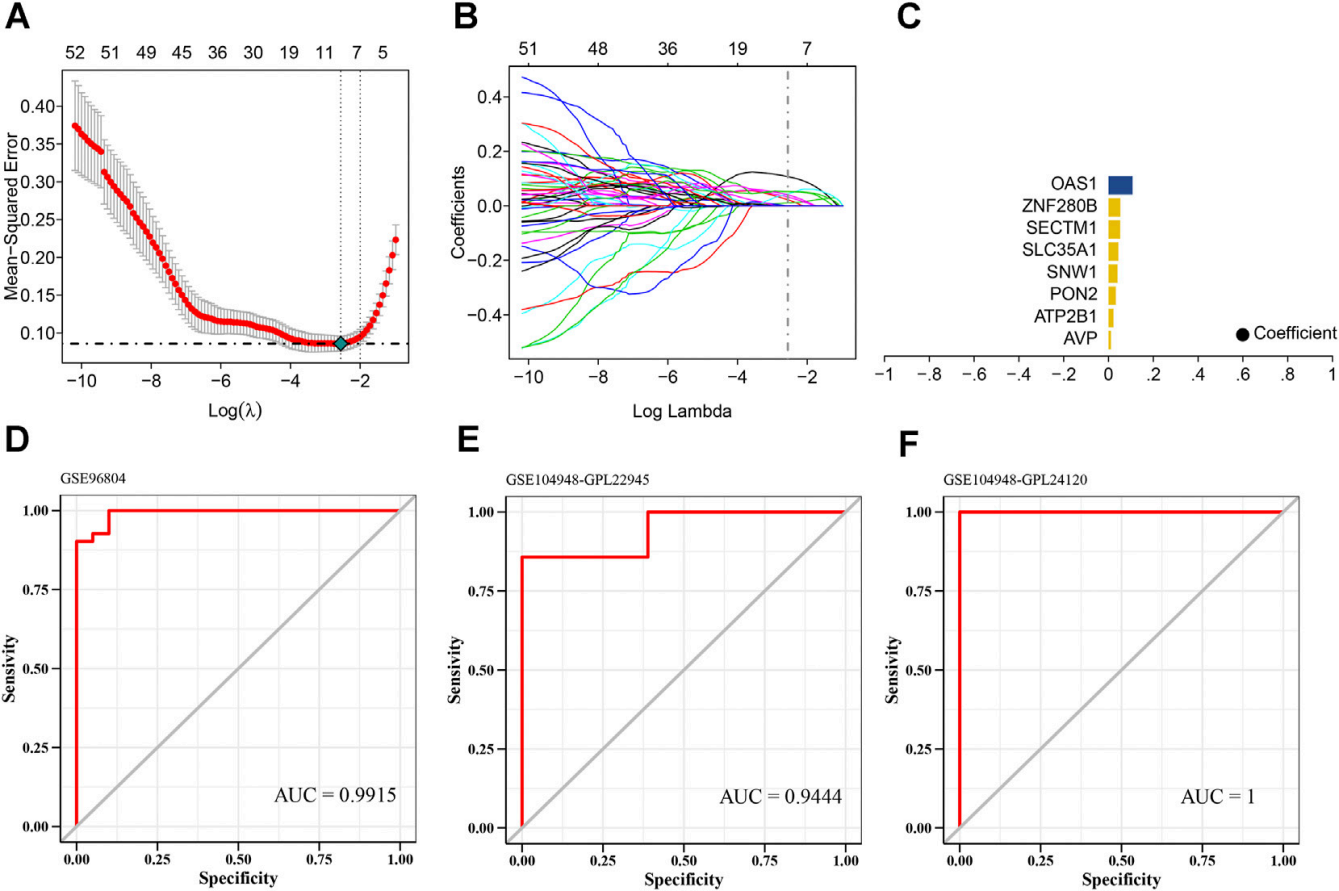
Screening of core genes in DKD. **(A)** Ten cross-validations of tuning parameter selection in the LASSO model to determine the minimum lambda value. **(B)** Distribution of LASSO coefficients for differential genes. **(C)** Coefficients of LASSO genes. **(D–F)** ROC curves of the eight LASSO genes in the training and validation sets. The areas under the AUC curve are all >0.9 and the model has good predictive performance.

Dataset GSE96804 was defined as the training set, while datasets GSE104948-GPL22945 and GSE104948-GPL24120 were defined as the validation sets. We selected the candidate genes for modeling in previous steps for feature screening through LASSO regression. The results of the LASSO regression identified eight genes as characteristic genes of DKD. Using these core genes, we performed follow-up studies and built a prediction model (Figures 5A–C). The model formula is: RiskScore = AVP x 0.0109739762830633 + ATP2B1 × 0.0224961639594229 + PON2 × 0.032598603699068 + SNW1 × 0.0403490710844676 + SLC35A1 × 0.0440058992110305 + SECTM1 × 0.0521504470451128 + ZNF280B × 0.0528500171681807 + OAS1 × 0.0521504470451128 + ZNF280B × 0.0528500171681807 + OAS1 × 0.0521504470451128. The results showed the good diagnostic performance of the prediction model based on eight genes, with an area under the AUC curve of 0.9915 (Figure 5D). We further used the GSE104948-GPL22945 and GSE104948-GPL24120 datasets as validation sets. As an external dataset for further validation of the diagnostic model, the results showed that the model has strong stability. The areas under the AUC curves of GSE104948-GPL22945 and GSE104948-GPL24120 were 0.9444 and 1, respectively (Figures 5E, F).

### 3.4 Protein interactions between markers

**FIGURE 6:**
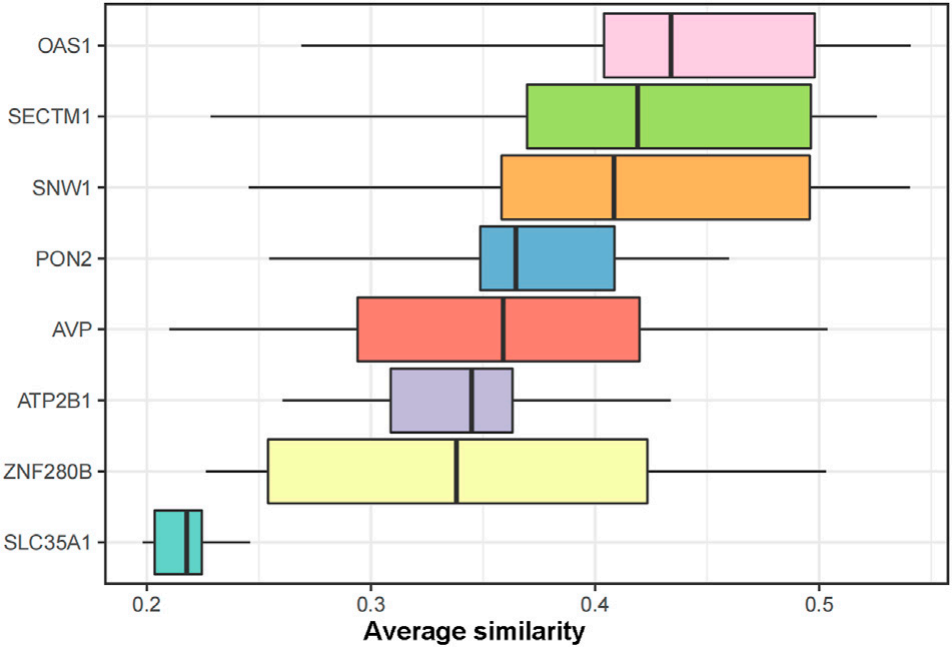
GO functional similarity ranking of the LASSO genes. The similarities in gene function distributions are summarized as boxplots. The line in the middle of the box represents the median, while the upper and lower boundaries show the upper and lower quartiles, respectively. The top three proteins with average functional similarity were defined as the core genes.

To further identify the core genes playing key roles in DKD, we performed a lasso analysis of 128 genes with |MM|>0.8 and |GS|>0.5 in the turquoise module of the WGCNA analysis. The results revealed eight lasso genes. The semantic similarity and geometric means of these eight genes between BP, CC, and MF were calculated using the mgeneSim function to obtain the final score. Finally, the DKD signature genes were ranked according to the average functional similarity relationship between the proteins. The results showed that SNW1, SECTM1, and OAS1 were the top three proteins in DKD. Therefore, we defined these three genes as the core genes of DKD and conducted follow-up studies (Figure 6).

### 3.5 Immune infiltration analysis

**FIGURE 7:**
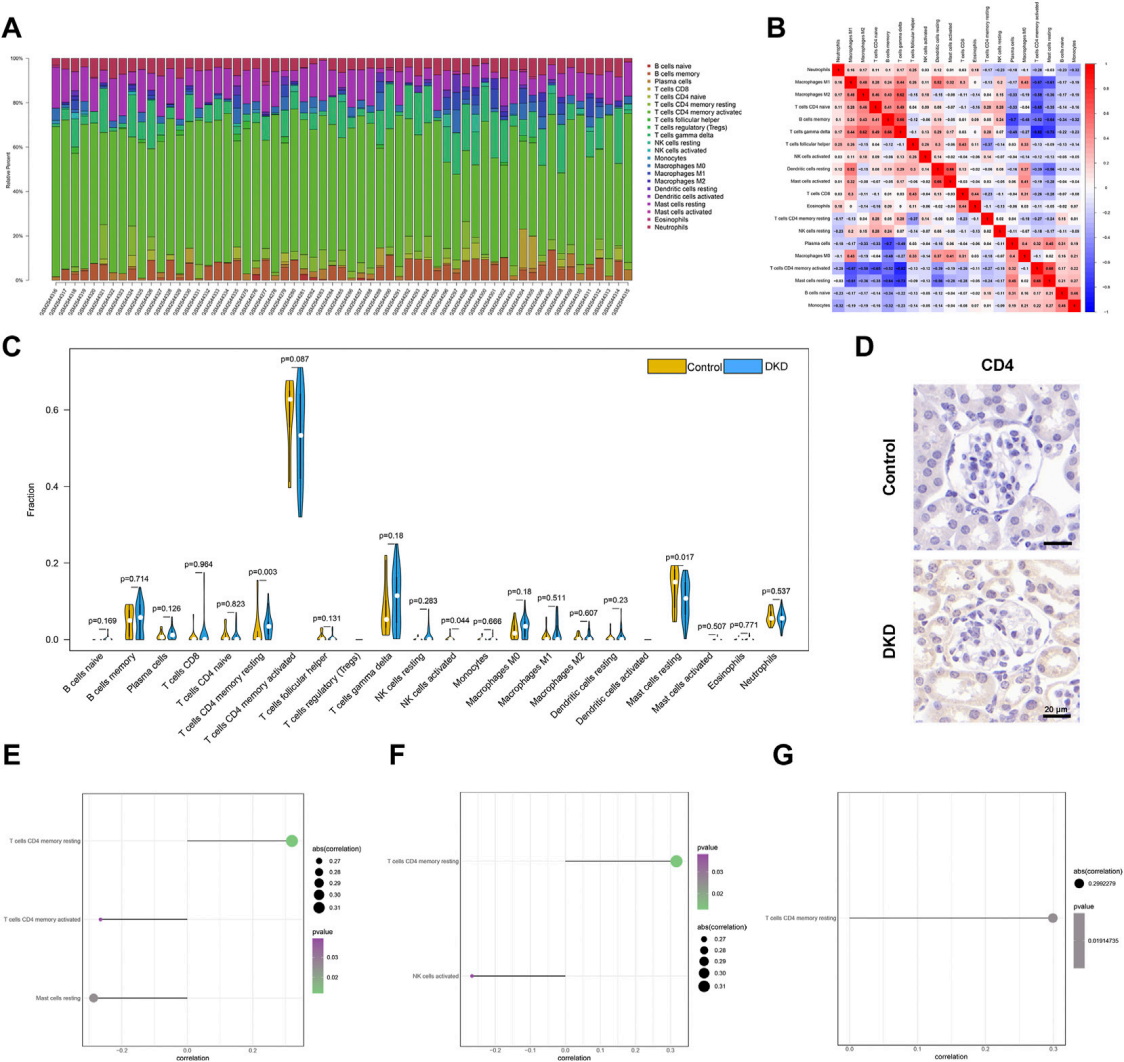
Immune infiltration of all samples. **(A)** Relative percentages of 22 immune cell subsets across all samples. **(B)** Pearson correlations between 22 immune cells. Blue and red indicate positive and negative correlations, respectively. **(C)** Differences in immune cell content between normal patients (yellow) and patients with DKD (blue). *p* < 0.05 is considered statistically significant. **(D)** Representative IHC staining of CD4 in the control and DKD groups. **(E–G)** Spearman correlations of OAS1, SECTM1, SNW1 gene expression and immune cell content.

The microenvironment is mainly composed of immune cells, extracellular matrix, growth factors, inflammatory factors, and special physical and chemical characteristics, which significantly affect the diagnosis of diseases and the sensitivity of clinical treatment. By analyzing the relationship between core genes and immune infiltration in the dataset, the underlying molecular mechanisms by which core genes affect DKD progression were further explored. The content and interactions between immune cells are shown in Figures 7A,B. The results showed that compared to unaffected patients, patients with DKD showed significantly higher levels of resting CD4 memory T cells and a lower number of resting Mast cells (Figure 7C). Immunohistochemical analysis showed increased CD4 expression levels in DKD mice (Figure 7D). The three core genes were strongly correlated with immune cells (Figures 7E–G).

### 3.6 Gene set enrichment analysis

**FIGURE 8:**
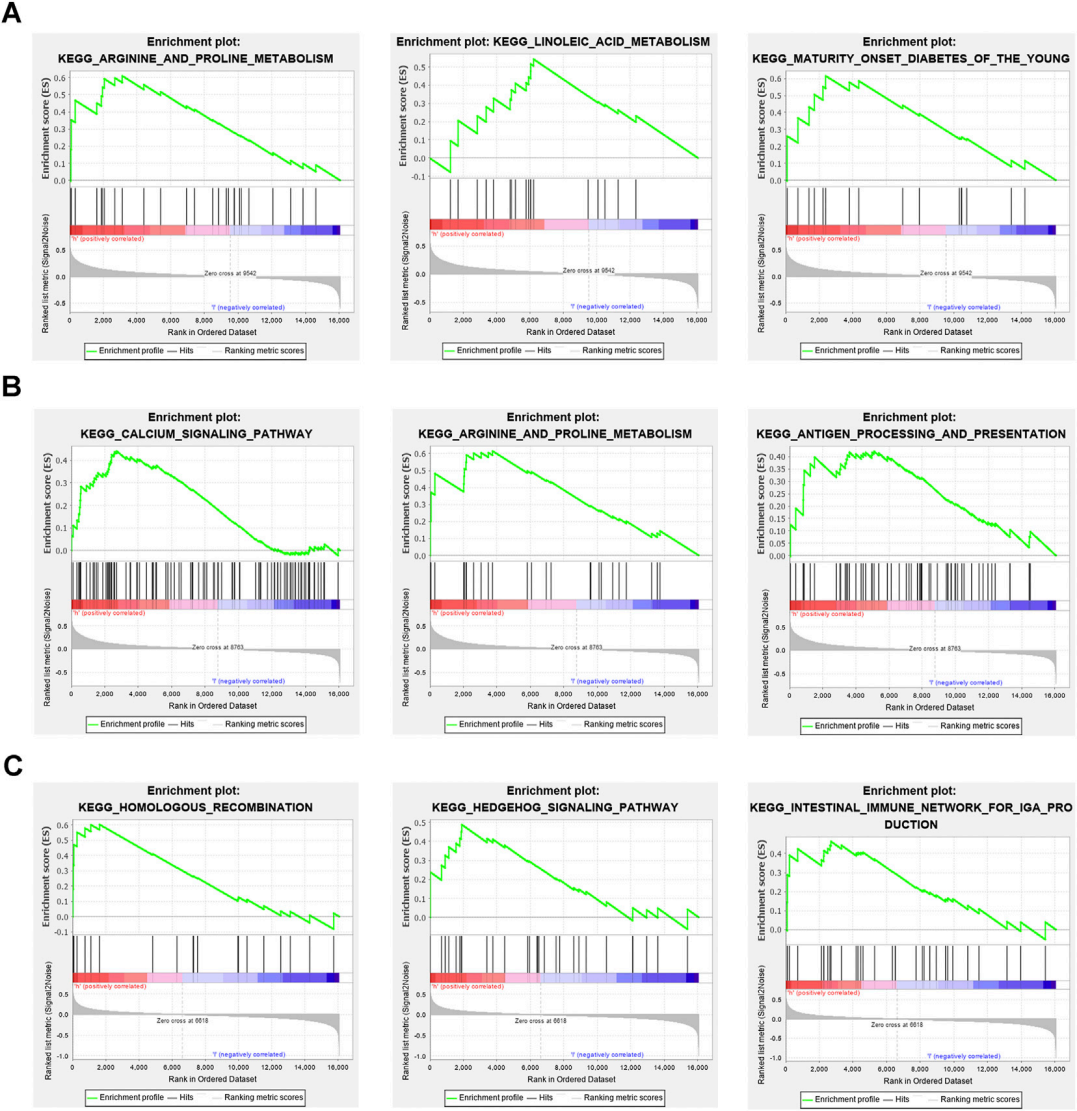
GSEA enrichment analysis of three core genes **(A)** The main enriched pathways with high expression for OAS1 are ARGININE AND PROLINE METABOLISM, LINOLEIC ACID METABOLISM, and MATURITY ONSET DIABETES OF THE YOUNG. **(B)** The main enriched pathways of high expression for SECTM1 are CALCIUM SIGNALING PATHWAY, ARGININE AND PROLINE METABOLISM, and ANTIGEN PROCESSING AND PRESENTATION. **(C)** The main enriched pathways with high expression in SNW1 are HOMOLOGOUS RECOMBINATION, HEDGEHOG SIGNALING PATHWAY, and INTESTINAL IMMUNE NETWORK FOR IGA PRODUCTION.

We next studied the specific signaling pathways enriched by the three core genes and explored the potential molecular mechanisms of the core genes affecting DKD progression. The GSEA results showed that the main enriched pathways for high *OAS1* expression were ARGININE AND PROLINE METABOLISM, LINOLEIC ACID METABOLISM, and MATURITY ONSET DIABETES OF THE YOUNG (Figure 8A). The main enriched pathways for high *SECTM1* expression were CALCIUM SIGNALING PATHWAY, ARGININE AND PROLINE METABOLISM, and ANTIGEN PROCESSING AND PRESENTATION (Figure 8B). Finally, the main enriched pathways for high *SNW1* expression were HOMOLOGOUS RECOMBINATION, HEDGEHOG SIGNALING PATHWAY, and INTESTINAL IMMUNE NETWORK FOR IGA PRODUCTION (Figure 8C).

### 3.7 Correlation analysis between markers and diabetic kidney disease

**FIGURE 9:**
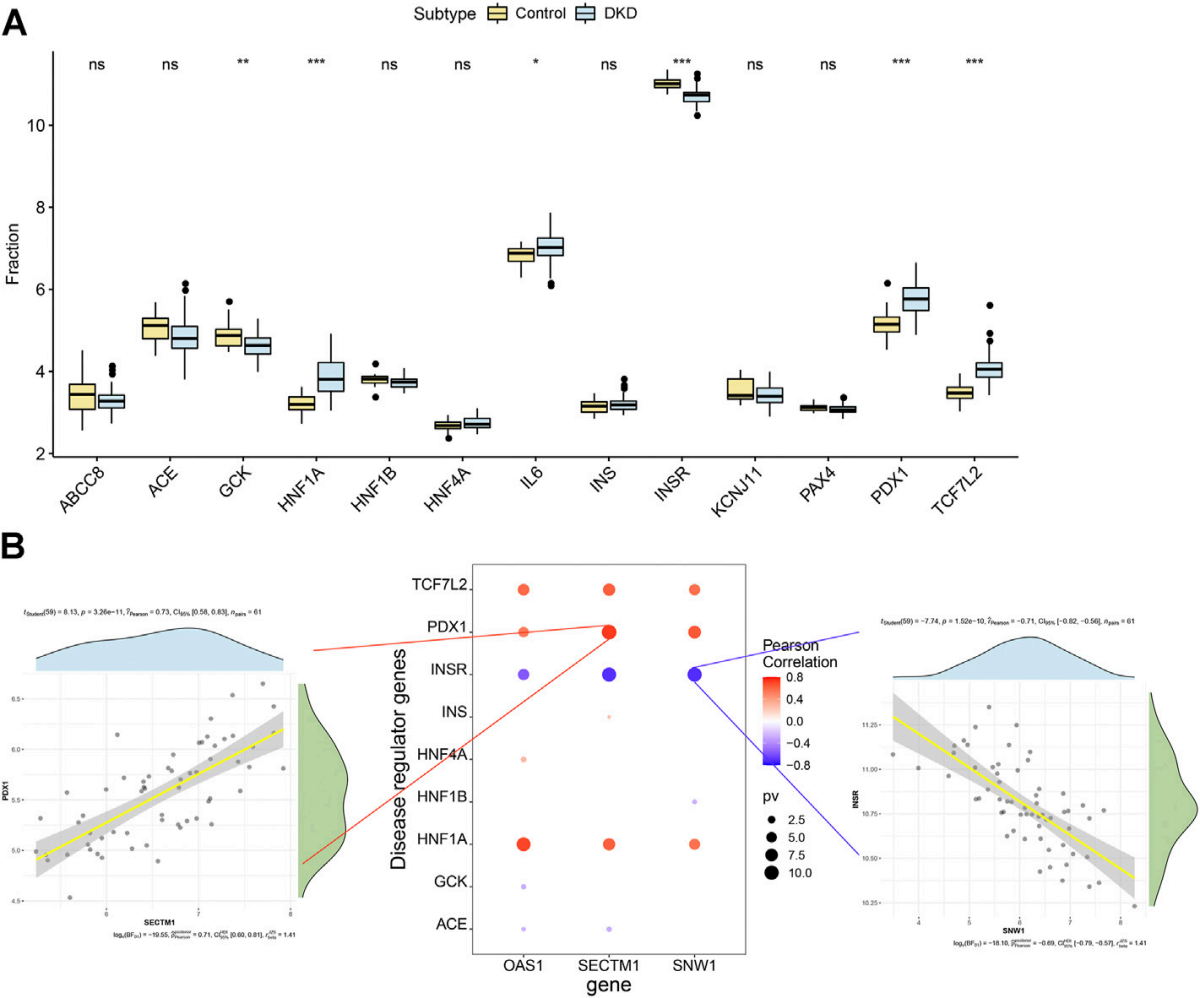
Correlation analysis of DKD-regulated genes. **(A)** Differences in the expression of DKD-regulated genes in control patients (yellow) and patients with DKD (blue). **(B)** Pearson correlation analysis of DKD-regulated and core genes. Blue and red indicate negative and positive correlations, respectively.

**FIGURE 10:**
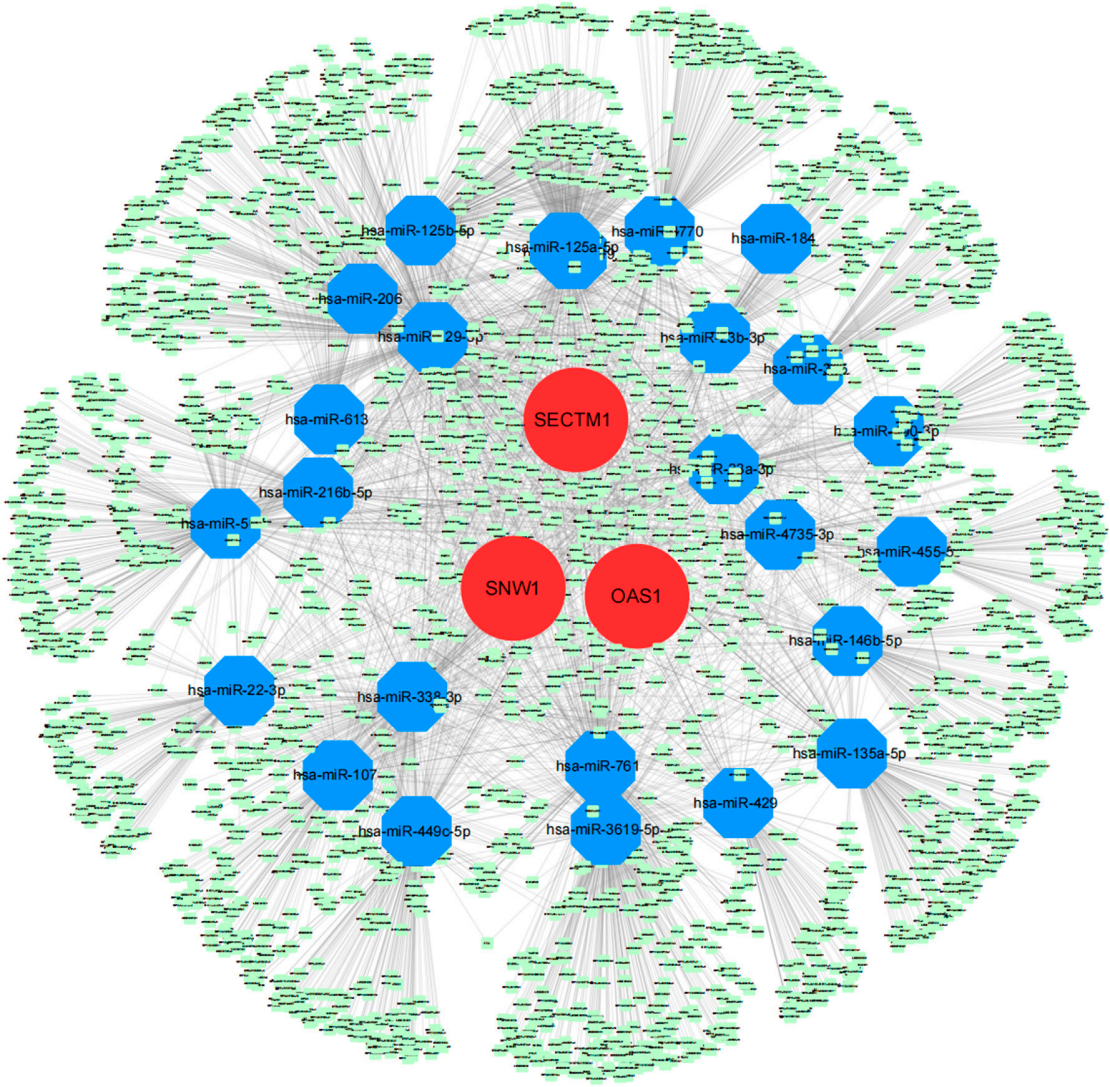
Core genes related to the ceRNA regulatory network. Red, blue, and green represent mRNA, miRNA, and lncRNA, respectively.

We obtained the disease-regulating genes of DKD through the Genecard database and performed differential analysis. The results showed that *GCK, HNF1A, L6, INSR, PDX1*, and *TCF7L2* differed significantly between the two groups of patients (Figure 9A). To explore the relationship between the core genes and DKD regulation, we performed a correlation analysis of the core and DKD-regulated genes. The results showed that *SECTM1* was significantly positively correlated with *PDX1* (Pearson r = 0.73), while *SNW1* was significantly negatively correlated with *INSR* (Pearson r = -0.71, Figure 9B). We also inversely predicted the targeted miRNAs of the three core genes through the mircode database and identified 104 miRNAs and 143 miRNA-mRNA relationship pairs. The 104 miRNAs were then inversely predicted through NPInter database, which revealed 2,690 lncRNAs and 4,549 miRNA-lncRNA relationship pairs. Combining these two results, we obtained 6,403 lncRNA-miRNA relationship pairs to construct the ceRNA network of core genes (Figure 10).

### 3.8 Modeling of diabetic kidney disease

**FIGURE 11:**
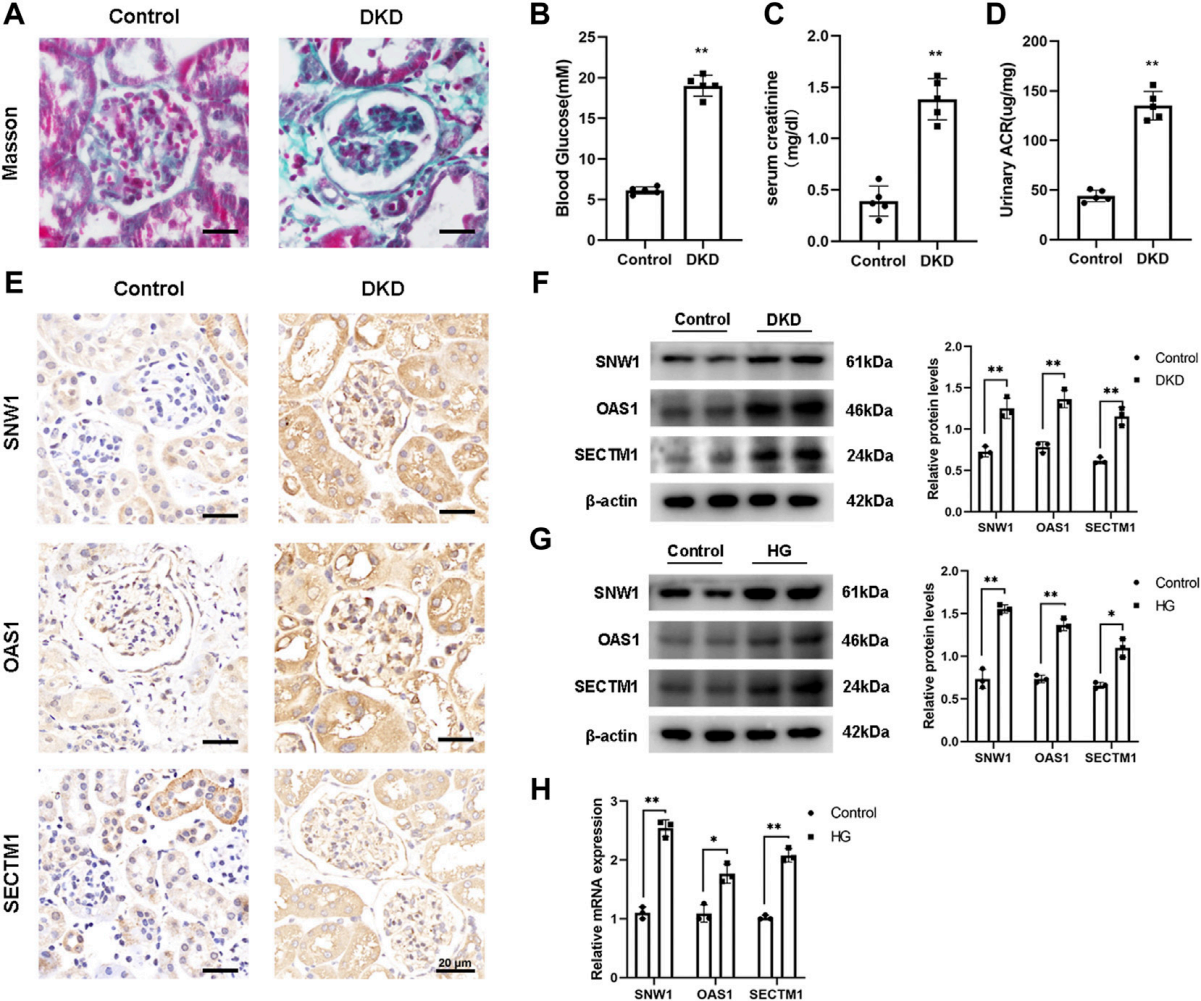
Validation of core gene expression. **(A)** Masson staining shows typical glomerular changes in DKD. **(B–D)** Detection of blood glucose and serum creatinine levels and urine albumin to creatinine ratio in mice. **(E)** Representative IHC staining of OAS1, SNW1, and SECTM1 in the control and DKD groups. **(F–H)** Detection of OAS1, SNW1, and SECTM1 expression by Western blot and qRT-PCR. ∗*p* < 0.05, ∗∗*p* < 0.01 vs. control.

The renal tissue of diabetic mice was observed by Masson staining. The results showed typical damage and severe collagen deposition in the renal tissue, indicating the successful construction of the diabetic mouse model (Figure 11A). In addition, the blood glucose level (Figure 11B), serum creatinine (Figure 11C), and urine albumin/creatinine levels of the diabetic mice were significantly increased compared to those in the control group (Figure 11D), further confirming the successful establishment of the diabetic mouse model.

### 3.9 Validation of *OAS1, SECTM1,* and *SNW1* as biomarkers of renal injury in diabetic kidney disease

Based on the results of bioinformatics analysis, we selected *OAS1, SECTM1*, and *SNW1* as candidate biomarkers. To examine their roles in DKD, we successfully constructed a DKD mice model and validated these genes *in vivo*. Immunohistochemical experiments showed decreased expression levels of *OAS1*, *SECTM1*, and *SNW1* in DKD mice (Figure 11E). Western blot analysis (Figure 11F) also showed significantly reduced expression levels of *OAS1*, *SECTM1*, and *SNW1* in DKD mice compared to normal controls. A DKD mesangial cell model was also established *in vitro*. After 24 h of culture in high-glucose medium, the protein levels of OAS1, SECTM1, and SNW1 were reduced compared to those in normal glucose medium (Figure 11G). Testing of treated HMCs for OAS1, SECTM1, and SNW1 mRNA expression by qRT-PCR showed substantially reduced expression of OAS1, SECTM1, and SNW1 mRNA in high-glucose-stimulated HMCs (Figure 11H). These results suggested that *OAS1*, *SECTM1*, and *SNW1* should be considered as biomarkers for the early detection of DKD in clinical trials.

## 4 Discussion

In the early detection of DKD, multi-factorial interventions targeting major risk factors (hyperglycemia, hypertension, dyslipidemia, and smoking) (Nathan et al., 1993; Gross et al., 2002; Mogensen, 2003) and the use of drugs with renoprotective effects (ACE inhibitors and/or ARBs) can reduce the progression of nephropathy (Viberti and Wheeldon, 2002; Mogensen, 2003). However, even with active treatment of known risk factors, some patients still develop DKD or eventually proceed to end-stage renal disease (Fioretto et al., 2006). Once in ESRD, patients require renal replacement therapy; however, the 5-year survival rate is <50% (Blumenthal, 2011). The main causes of DKD are the disturbance of glucose metabolism, oxidative stress, and altered renal hemodynamics (Wada and Makino, 2013). Nevertheless, the exact molecular mechanism is not clear and effective treatment options are lacking. Therefore, the identification of new targets is urgently needed to prevent the occurrence and development of DKD.

The rapid development of bioinformatics has helped us to explore the pathogenesis and related molecular pathways of DKD, and further seek potential new biomarkers of this disease. In this study, by analyzing differentially expressed genes (DEGs) in the GSE96804 datasets, LASSO regression identified eight characteristic genes (*AVP, ATP2B1, PON2, SNW1, SLC35A1, SECTM1, ZNF280B,* and *OAS1*) to construct a diagnostic model of DKD. This diagnostic model based on eight key genes distinguished healthy control (HC) from DKD samples with high accuracy (99.15%), strongly supported by the 94.52% AUC and 100% AUC in two separate external validation cohorts. Some of these eight genes have been confirmed to play important roles in the pathogenesis of DKD. AVP is a small peptide composed of nine amino acids that is synthesized in specific neurons in the paraventricular and supraoptic nucleus, stored in the neurohypophysis, and exerts different physiological effects by binding to different receptor subtypes (Rotondo et al., 2016). (Tahara et al. (2008)found that AVP can promote extracellular matrix (ECM) synthesis by regulating the secretion of TGF-β in rat mesangial cells (RMC), which leads to glomerulosclerosis in DKD. *PON2* is the oldest member of the paraoxonase family and plays an integral role in the control of oxidative stress, inhibition of apoptosis, and progression of various malignancies (Manco et al., 2021). Pinizzotto et al., (2001) demonstrated that polymorphisms in *PON2* were significantly associated with DKD independently of the traditional risk factors for type II diabetes (T2DM) In our study, metascape enrichment analysis revealed DEGs that may affect the development of DKD through a variety of signal pathways and targets in biological processes, mainly including unsaturated fatty acid biosynthetic process, positive regulation of receptor signaling pathways via JAK-STAT, carbohydrate metabolic process, protein processing in the endoplasmic reticulum, and prion diseases. Notably, the JAK-STAT signaling pathway plays important roles in regulating various pathophysiological processes, such as inflammation, homeostasis, cell proliferation, and apoptosis (Banerjee et al., 2017; Seif et al., 2017). The activity of the JAK-STAT pathway is enhanced in DKD animal models. Inhibition of this activity suppressed STZ-induced renal damage in DKD mice (Lu et al., 2009), while activation of the JAK-STAT signaling cascade can stimulate the excessive proliferation and growth of glomerular mesangial cells, which leads to DKD (Amiri et al., 2002).

Immune cell infiltration is significantly associated with the pathogenesis of metabolic disorders such as T2DM, obesity, and metabolic syndrome. Phenotypic changes in immune cells and inflammatory infiltration precede the development of metabolic disorders (Guzik and Cosentino, 2018). Accumulating evidence indicates the important role of immune cell infiltration and inflammation in the pathogenesis of DKD (Tesch, 2017). The present study identified the infiltration of 22 immune cell types in DKD by CIBERSORT. The DKD group showed a higher proportion of CD4 memory resting T cells and a lower proportion of resting mast cells. CD4^+^ T cells are central regulators in chronic metabolic inflammation, involved in the mediation of macrophages and other T and B cell-dependent inflammatory responses (Trifari et al., 2009). Orban et al. (2014) reported that CD4^+^ memory T cells are intimately involved in the pathogenesis of type Ⅰdiabetes (T1DM) and may be a potential immune marker for the diagnosis of T1DM. Lin et al. (2021) reported increased levels of CD4 memory resting T cells in diabetic neuropathy, which may be related to disease progression. Major histocompatibility complexes (MHCs) are related to the immune response, immune regulation, and the production of certain pathological conditions. Mast cells are a type of innate immune cell that can express MHC molecules (Agier et al., 2021). Mast cells are also functional negative regulators in T1DM and other autoimmune-related diseases (Geoffrey et al., 2006; Fehervari, 2018). Okoń and Stachura (2007) demonstrated that mast cells are closely related to serum creatinine and urea levels in patients with DKD. The results of our study showed a comparatively large proportion of mast cells in the HC samples, possibly due to a potential limitation of the CIBERSORT algorithm. The high ratio of CD4^+^ memory T cells in patients with DKD makes the ratio of other immune cells, including mast cells, appear relatively low. Meanwhile, we further confirmed the correlation between three core genes (*OAS1, SECTM1,* and *SNW1*) and immune cell infiltration. Our results suggested that the three core genes were consistently associated with immune infiltration and were closely related to resting memory T cells, activated T cells, resting mast cells, and activated NK cells. These results provide further evidence of the important role of the core genes and immune infiltration in DKD progression.

Among these core genes, *OAS1* encodes a protein of the 2’, 5’-oligoadenylate synthase family, a group of enzymes that play an important role in innate antiviral defense (Justesen et al., 2000). Field et al. (2005) proposed that *OAS1* activation may promote β-cell apoptosis, thereby enhancing susceptibility to T1DM. Pedersen et al. (2021) showed upregulation of *OAS1* in the islets of T1DM. *OAS1* is also relevant in multiple sclerosis, and hepatitis C virus infection (García-Álvarez et al., 2017; O'Brien et al., 2010). *SECTM1* is a type I transmembrane glycoprotein, located in the Golgi apparatus, with transmembrane and soluble forms (Slentz-Kesler et al., 1998). Targeting SECTM1-CD7 interactions may be used to prevent allogeneic T cell responses, autoimmune diseases, and IFN-γ (Wang et al., 2012). *SNW1* is a multifunctional protein that plays a role in disease through direct protein interactions, mRNA splicing regulation, or transcriptional control (Verma et al., 2019). SNW1 overexpression is a marker of increased cancer aggressiveness and has been confirmed in a variety of cancers, including breast cancer, hepatocellular carcinoma, and prostate cancer (Liu et al., 2013; Liu et al., 2014; Höflmayer et al., 2019). Our *in vivo* and *in vitro* experiments demonstrated that the relative transcript levels of core genes showed the same expression trends as those in the bioinformatics analysis, with significantly increased DKD expression. Moreover, GSEA revealed specific signalling pathways of the three core genes that may participate in the development of DKD, providing a direction for further exploration of DKD pathogenesis.

In this study, we identified regulatory genes correlated with DKD through the Genecard database. Six genes (*GCK*, *HNF1A*, *L6*, *INSR*, *PDX1*, and *TCF7L2*) differed significantly between the HC and DKD groups. *PDX1* (pancreatic and duodenal homeobox 1) and *INSR* (insulin receptor) are known genes that modulate DKD. *PDX1*, one of the earliest active pancreatic transcription factors, directs beta-cell differentiation and physiological insulin gene transcription (Zhu et al., 2017). *PDX1* knockout resulted in early DKD features such as glomerular basement membrane (GBM) thickening and glomerular hypertrophy in the zebrafish pronephros, while *PDX1* overexpression inhibited the progression of diabetic kidney damage (Wiggenhauser et al., 2022). *INSR* encodes the insulin receptor and plays an important role in the insulin signaling pathway (Payankaulam et al., 2019). Gatica et al. (2013) reported that INSR is highly expressed in the kidney tissue of patients with DKD, suggesting its role in the development of DKD. Our analysis of the correlations between three core genes and DKD regulatory genes revealed intimate connections among them. While the mechanism of the three core genes and DKD regulatory genes in the pathogenesis of DKD is unclear, the above evidence implies that the related regulatory genes of DKD may participate in the development of DKD by regulating the expression of the core genes. In addition, we also constructed a ceRNA network of core genes by combining the NPInter and miRcode databases. Although less information is reported on the association of these miRNAs and lncRNAs with the progression of DKD and other renal diseases, these lncRNAs, miRNAs, and mRNAs may play potential and important roles in the regulation of renal function.

We analyzed the DEGs related to DKD in the GEO database based on only eight signature genes that could be used to construct a diagnostic model with high accuracy. We discovered three core genes (*OAS1*, *SECTM1*, and *SNW1*) that may be related to the pathogenesis of DKD and further revealed that they may influence DKD progression through various biological functions and pathways. These findings provide new ideas for the pathogenesis and treatment of DKD.

## Data Availability

The datasets presented in this study can be found in online repositories. The names of the repository/repositories and accession number(s) can be found in the article/Supplementary Material.
