# Supplementary material for: Identification and validation of key biomarkers for the early diagnosis of diabetic kidney disease
Source: Front Pharmacol. 2022 Aug 22;13:931282. doi: 10.3389/fphar.2022.931282 (PMC9441656; doi:10.3389/fphar.2022.931282)
Supplement: Supplementary file 1 [file Table1.DOCX]

TABLE S1

Primers used in qRT-PCR experiments

| Gene | Forward primer (5′-3′) | Reverse primer (5′-3′) |
| --- | --- | --- |
| SNW1 | CCTGGTTCCAAAGGAGGTTATG | GCGGCGACCTTCTGTGATA |
| OAS1 | AGTTGACTGGCGGCTATAAAC | GTGCTTGACTAGGCGGATGAG |
| SECTM1 | CTGGAGGTTTCAGGTGCAGAA | GAACATGACCAGAGCGACCA |
| β-actin | CGTTGACATCCGTAAAGAC | TAGGAGCCAGGGCAGTA |
